# Epigeneti-What? Approaches on Translating Research for Primary Breast Cancer Prevention

**DOI:** 10.3389/fonc.2019.00267

**Published:** 2019-04-12

**Authors:** Evan K. Perrault, Grace M. Hildenbrand, Robert G. Nyaga

**Affiliations:** Brian Lamb School of Communication, Purdue University, West Lafayette, IN, United States

**Keywords:** breast cancer, translation, prevention, health communication, epigenetics

## Abstract

In fiscal year 2017, the National Cancer Institute devoted more than a half billion dollars to breast cancer research. Since 2012, the total investment has been more than $3 billion. Despite this significant investment, breast cancer still has no known immediate causes as it generally develops over the life course. Therefore, research is unable to provide the public any sort of magic bullet, or conclusive link between certain environmental exposures and the development of breast cancer later in life. What research is only able to report are likelihoods—possible links—things people might want to consider avoiding or doing in their everyday lives to reduce their future risks of developing breast cancer. This abundance of rigorously performed, albeit causally inconclusive, research focused on “plausible” links poses a challenge for health communicators who are tasked with seeking to find ways to translate this science into advice that people can act upon today. However, if society must wait for the science to provide 100% conclusive evidence before anyone ever takes action, how many lives could have been saved in the interim? Therefore, we advocate a two-pronged approach to translating scientific findings regarding environmental exposures and breast cancer prevention: a bottom-up approach—focused on informing the lay public and individuals, while simultaneously performing a top-down approach—focused on influencing policymakers. The current perspective analyzes the strengths and weaknesses to both of these approaches, and encourages scientists to work closely with health communicators to develop theoretically-driven strategies to drive positive changes over time.

## Introduction

The purpose of science is often described as a pursuit of new knowledge and understanding. This pursuit is awarded billions of publicly-funded dollars each year by governments around the globe. While much of this research is likely to find a home in pay-walled peer-reviewed publications for other scientists to read, very little is likely to make its way directly to the public—those who actually funded this research in the first place. New knowledge—knowledge that if placed in the right hands could potentially save lives—does no one any good if it sits on a shelf and is not shared with others. Given the enormous economic burden that cancer, especially breast cancer, places on society (1), it is imperative research findings that may provide a window into preventing cancers from ever occurring in populations be translated for public consumption.

One of these areas of research receiving significant support is the area of epigenetics, the study of how chemicals present in foods and drinks we all consume may alter gene expression through hormonal disruptions and increase breast cancer risks (2). While the term “epigenetics” has received a lot of attention in the media, most lay individuals do not adequately understand the term (3). This could be because the term is not all that easy to explain in a simple sound bite—often using highly complex multisyllabic vocabulary to discuss how it actually effects a person's biology [e.g., “methylation epigenetic modification;” (4)]. Unlike simply translating information from one language to another using electronic translators or dictionaries, there is no magic elixir for translating complex scientific information into ideas that are easily digestible for the lay public or policymakers to act upon.

Translating the latest epigenetic research simultaneously for these two primary target audiences is essential if we ever hope to achieve zero prevalence of breast cancer in society. Individuals have the power to make localized changes within their own personal spheres, while policymakers have the power to change entire societies through the enactment of new laws. However, reaching and changing these two very different groups will require shifts in standard tactics and strategies, true interdisciplinary collaborations between the biological and social sciences, and likely a good dose of patience. The following perspective labels these approaches bottom-up (focusing on reaching individuals directly) and top-down (focusing on reaching policymakers), and discusses the unique benefits and challenges to embarking on each of these approaches.

## Individual Focus: The Bottom-Up Approach

### Going Beyond Traditional News Media to Reach Individuals

General news outlets have commonly been utilized as a popular means to help scientists spread their research beyond the walls of their laboratories to reach individuals in their homes. This is because, for usually very little cost and effort, institutions can write press releases about new research that sometimes get picked-up by the media, and have the potential to reach large segments of the lay public (5). However, while these news outlets can serve as a means to get stories out to large audiences, this dissemination often comes with a loss of message fidelity.

Only 28% of Americans state they think news outlets get science facts right most of the time (6). News stories on genetic research about cancer tend to not be as accurate as the press releases from which they obtain their information (7), and often shy away from reporting on cancer prevention-related topics (8). For example, only about 4% of news stories about breast cancer analyzed by Atkin et al. (8) covered environmental hazards such as risks connected to chemical contaminants. There are a host of potentially carcinogenic chemical compounds found in everyday household products that could be related to increased breast cancer risk later in life; for example: bisphenol A (BPA) found in plastics, butyl benzyl phthalate (BBP) used in food packages and cosmetics, and perfluoroactanoic acid (PFOA) which is contained in some industrial and consumer goods (9). These chemicals often consist of a confusing string of jargon for both journalists and the lay public to comprehend, making it daunting to determine which products to avoid, but more importantly the epigenetic science behind why individuals may want to refrain from using them in their daily lives.

Therefore, it is no wonder why the media tend to overgeneralize results from epigenetic scientific studies (e.g., stating cause-and-effect relationships) that instead should be approached with more nuance and tentative language (10). “The simplification that is often necessary for good, clear journalism can foster inferences that go far beyond the original observation from which the inferences were drawn” (10; p. 4). This is why medical professionals and researchers should actively seek collaborations with health-beat reporters to help them to see which risks should receive attention in stories, and also potentially serve as fact-checkers to help ensure the accuracy of the information ultimately shared with the public (11).

However, instead of relying on news personnel to essentially act as mediators between scientists and the lay public—who may inadvertently get the science incorrect—breast cancer researchers should be seeking to reach the public directly to educate them on the latest scientific research to reduce their breast cancer risks.

### Communicating Scientific Uncertainty via the Precautionary Principle

Breast cancer researchers might be hesitant to advocate that individuals make various changes in their lives to reduce their breast cancer risks simply because no research regarding chemical influences on breast cancer and the human epigenome is 100% certain. While true, scientists need to realize that the science will never be 100% conclusive, and should therefore frame their research to the public around the precautionary principle. In other words, even though breast cancer prevention research continues to be ongoing, with findings that will likely never be able to truly find cause-and-effect relationships, letting the public at least know these findings may still make a difference by saving lives at an individual level (12). Research indicates that communicating this scientific uncertainty does not negatively influence the public's interest in science or perceptions of the trustworthiness of scientists (13), suggesting it is not detrimental for scientists to express that findings are uncertain.

### Finding Allies in Health Communication

Scientists seeking ways to communicate their research to the lay public should look no further than colleagues they may have across campus in the liberal arts or humanities within the discipline of health communication. Health communicators are social scientists trained in the study of using evidence- and theory-based approaches to effectively change individuals' knowledge, attitudes and behaviors surrounding health topics (14). For example, projects stemming from health communicators embedded within the National Cancer Institute funded Breast Cancer and Environment Research Program (BCERP) found that higher literacy-level translated research regarding progesterone's potential impact on breast cancer was actually more effective at increasing the public's perceptions of risk than messages translated to a lower literacy-level (15). Silk et al. (16) also found that the public does desire some level of scientific complexity in breast cancer prevention messaging in order to help them better understand the relevance of the research to their daily lives.

Health communication scholars are able to utilize a large toolbox of formative research skills (e.g., survey design, in-depth interviewing, data analysis) in order to determine which elements of theory should be emphasized in messages targeted to the lay public (17). For instance, Smith et al. (18) conducted research guided by the Heuristic Systematic Model to determine the way capability, motivation, and different types of processing result in particular beliefs and attitudes about environmental breast cancer risks from PFOA. Such theoretical guidance is essential to not only guarantee that resources are well spent, but also to ensure the public is motivated by messages that are developed to make well-informed decisions.

### Strategies for Communicating Environmental Risk Factors

Developing highly tailored campaigns and interventions for specific target audiences is likely to yield the most promising results in changing the publics' knowledge, attitudes, and behaviors toward potential environmental breast cancer risks (19). When communicating uncertain scientific findings to the public, one effective strategy is to present multiple claims and then state how many experts believe each claim, generating perceptions of certainty about a scientific claim (20). If a breast cancer risk message conveys the number of scientists who believe there is a need to take environmental risks seriously, this might motivate members of the public to engage in precautionary behaviors such as avoiding consumer products that contain chemical toxins.

Scientists must also move beyond scholarly outlets to reach lay audiences (14). While members of the public mainly obtain scientific information from the media, they rely on multiple sources, using both online and traditional communication channels (21). Thus, a majority of health campaigns make use of multiple channels in order to reach the greatest number of people (22). In translating scientific information, communicators should select channels that are easily accessible by the public, and that capture their attention (23). Personal communication in the form of interpersonal influence is a valuable supplement to mass communication and a strong contributor to behavior change. Campaign managers would be wise to find key influencers in particular communities and persuade them to influence others (24). Another strategy that is effective at informing a lay audience about scientific information is using website videos to discuss possible environmental risks for breast cancer (25). Channels should be selected based on preferences of the target audience—not on the personal preferences of scientists.

### Strengths and Weaknesses of a Bottom-Up Approach

One major strength of developing communication geared directly toward individual-level behavior change—compared to policies—is that individual-level change is rarely controversial. No one gets outraged when individuals decide to voluntarily change their diets, purchase behaviors, or exercise habits. Communication targeting individuals is also likely to lead to quicker changes (e.g., changing knowledge, attitudes, or behaviors) than communication seeking policy change, which can take years to pass and even longer to enact. However, one key weakness of this bottom-up approach is that these strategies tend to have only small to medium effects on influencing knowledge, attitudes, and behaviors (22). Reaching all members of a population is difficult, if not impossible, and even if people receive a message, this does not mean the amount of exposure was sufficient to fuel behavior change.

Therefore, a multi-pronged approach is advocated. While attempts are developed to help change the public at the individual level, scientists should simultaneously be working to change the minds of lawmakers to develop policies that could allow for a much more substantial impact on populations.

## Focusing on Policymakers: A Top-Down Approach

Beyond communicating cancer research effectively to individuals and the public, there is also a need to anchor interventions on policies that protect their safety from carcinogens, and ensure penalties for industries whose products expose the public to cancer-related risks. The recent revelation that Johnson & Johnson may have known for decades that its talcum baby powder may have contained asbestos (26) showcases a need for policies to control potentially carcinogenic substances and highlight objective research that is devoid of potential influence by profit-driven industry players. To achieve such goals, it is important for scientists and policymakers to work together to formulate evidence-based cancer policies.

However, so far, policymakers and scientists seem disconnected (27, 28), and often do not share the same priorities and values (29–31). These tensions undermine the role of research in policymaking and attest to the need of dialogue between the two groups as a way of bolstering the progress made so far in the war on cancer.

### Why Should Breast Cancer Researchers and Policymakers Work Together?

It is important for the policymaking and scientific communities to work closely together to ensure robust policies that address salient issues associated with breast cancer, such as exorbitant treatment costs, reduction of quality life years and loss of productivity due to employment disability, missed work days, and days spent in bed (32). This is particularly important because by 2020, the loss of present value of lifetime earnings (PVLE) due to cancer is estimated to be $147.6 billion, with breast cancer leading in the loss of PVLE among women below 55 years of age (33). Additionally, the caregiving costs associated with cancer in 2000 were estimated at $232.4 billion and are expected to rise to $308 billion by 2020 (33). In the non-elderly population, breast cancer has the second highest adjusted annual economic burden estimated at $14,167 after colorectal cancer (32). These high costs associated with breast cancer treatment point to the need for epigenetic breast cancer prevention researchers to begin to advocate for policy changes that could lead to substantial benefits for populations decades and centuries into the future.

### Effective Communication of Cancer Research to Policymakers

To enhance the effectiveness of breast cancer prevention research in informing policymaking, it is imperative that scientists communicate their research findings in a way that captures the attention of policymakers because some of them, especially legislators, are inundated by the volume of policy-related information they receive (34–36). One effective way to do this may not be by reaching out directly to policymakers, but instead by reaching them indirectly through the mass media—a strategy known as media advocacy (37). The goal of media advocacy is to use a mix of both paid (e.g., advertisements) and unpaid media (e.g., PSAs, grass roots organizing) to set the media's agenda and get a topic wide attention. When the topic is on the media's agenda (e.g., it is a lead story for multiple days), policymakers are sure to pay attention. For example, individual researchers, or organizations like the IBCN, could start by writing a series of Op-Eds regarding policy changes that could have an impact on reducing breast cancer (38). Researchers could also come out with a series of policy statements, and generate news coverage through manufactured press events (e.g., rallies, community demonstrations) that would appeal to news outlets. To enhance their persuasiveness, researchers could also make arguments for the wider relevance of their research by extrapolating their findings across states and/or countries (30, 39) thereby helping policymakers to understand the potential impact of their research and how novel policies could help to ameliorate the effects associated with breast cancer.

Researchers may also want to initially aim small in trying to achieve policy changes. Changes at the local level (e.g., city, county) are likely to take place much quicker than at a national level. These local level changes could also ultimately lead to much more significant changes. For example, products required in California through Proposition 65 to carry a message stating they contain chemicals known to the state to cause cancer, can oftentimes be found across the United States—thereby extending the impact of this local policy. Similarly, researchers could strive to enact a policy at one elementary school, one university, or in one city, banning the sale of certain foods or products that contain chemicals known to detrimentally effect the epigenome. This ban could then have ripples across the supply chain in a region, thereby essentially eliminating a potentially hazardous product in more than just the municipality with the ban.

Overall, to bridge the gap between scientists and policymakers, it is necessary for these two groups to build relationships and create avenues for effective deliberations. This participatory approach might encompass scholars inviting policymakers to their classes, or policymakers inviting researchers to their forums to offer input on cancer policies (28). Although researchers and policymakers have working differences, when policymakers are faced with dilemmas, they turn to academics for alternative agendas (40). Therefore, the role of scholars in generating policy issues cannot be underestimated. Kingdom (40) also advises that researchers join policy communities, which are composed of specialists in a given policy area. Such communities are important because they can help scientists to build networks with advocacy groups, enhance their understanding of the information needs of policymakers, and have opportunities to learn health policy language (34, 41).

### Strengths and Weaknesses of a Top-Down Approach

The clear strength of the top-down approach is that changing policy is likely to have long-lasting effects on society. For example, enacting policies to fluoridate public water supplies has led to significant reductions in cavities over the last 70 years, and is cited as one of the top-10 public health achievements of the 20th century (42). However, changing policies is likely to be a much lengthier endeavor than seeking to change individual behaviors through campaign efforts. Therefore, advocating policy change should be seen as part of a comprehensive strategy—alongside individual behavior change—to achieve breast cancer prevention.

## Conclusion

In conclusion, neither the bottom-up nor top-down strategy should be used in isolation. While utilizing the bottom-up approach researchers are likely to see effects rather quickly, but these effects will likely be limited to small pockets of populations, and potentially not very long lasting. Utilizing the top-down approach is likely to yield much larger dividends, but it also comes with a much longer time commitment, and no guarantee of success after years of advocacy work. To maximize return-on-investment, breast cancer prevention researchers should seek to translate their findings simultaneously along both of these routes, and seek guidance from interdisciplinary colleagues trained in their intricacies—those in the health communication discipline.

If researchers truly want to advance knowledge, part of that advancement has to be translating and disseminating their work to the public to help them act on it in meaningful ways. Until breast cancer prevention researchers are ready to work comprehensively and share resources across disciplinary boundaries with those in communication, it is likely researchers' advancement of knowledge will stop at the peer-reviewed publication of their work—relegated to a dusty shelf or seldom used online depository—and society will potentially be no better off for it.

## Author Contributions

All authors listed have made a substantial, direct and intellectual contribution to the work, and approved it for publication.

### Conflict of Interest Statement

The authors declare that the research was conducted in the absence of any commercial or financial relationships that could be construed as a potential conflict of interest.
